# Role of Damage Control Surgery in Perforated Diverticulitis Management: A Systematic Review

**DOI:** 10.7759/cureus.89740

**Published:** 2025-08-10

**Authors:** Samyuktha Harikrishnan, Sanathanan Neelakantan Ramaswamy, Yashasvi Agarwal, Nehal K Bhatt, Shalvin Chand, Manvitha Bendagiri Matam, Lubna Mohammed

**Affiliations:** 1 College of Medicine, Gulf Medical University, Ajman, ARE; 2 Internal Medicine, Government Erode Medical College and Hospital, Erode, IND; 3 Internal Medicine, Sir H.N. Reliance Foundation Hospital and Research Centre, Mumbai, IND; 4 Internal Medicine, Jawaharlal Nehru Medical College, Belagavi, IND; 5 Internal Medicine, Pramukhswami Medical College, Anand, IND; 6 College of Medicine, University of Fiji, Lautoka, FJI; 7 College of Medicine, Gandhi Medical College, Secunderabad, IND; 8 Nephrology, St. Helier Hospital, London, GBR; 9 Internal Medicine, Dr. V.R.K. Women’s Medical College, Hyderabad, IND

**Keywords:** anastomosis, damage control surgery, perforated diverticulitis, stoma, trauma surgery

## Abstract

Perforated diverticulitis is a critical surgical emergency that demands immediate attention and intervention. Damage control surgery (DCS) has emerged as an alternative to traditional approaches for managing physiologically unstable and critically ill patients. This systematic review includes 16 articles published between 2015 and 2025, covering randomized controlled trials, observational cohorts, case reports, and guidelines. The inclusion and exclusion criteria focused on human studies published in English that addressed managing perforated diverticulitis with DCS. The Preferred Reporting Items for Systematic Reviews and Meta-Analyses (PRISMA) 2020 guidelines were used to guide article selection, which was further refined using standardized appraisal tools such as A Measurement Tool to Assess Systematic Reviews 2 (AMSTAR 2), the Newcastle-Ottawa Scale (NOS), Joanna Briggs Institute Checklists (JBI), the Cochrane Risk of Bias (RoB) tool, the Appraisal of Guidelines for Research and Evaluation II (AGREE II), and the Checklist for Reporting Results of Internet E-Surveys (CHERRIES). This review highlights DCS as an effective approach for managing perforated diverticulitis. Patient management with DCS showed significant improvement compared to traditional methods such as Hartmann’s procedure. Physiological stabilization reduced complications such as stoma formation and improved anastomosis rates, as supported by the included studies. The findings suggest that DCS reduces morbidity and mortality, lowers the need for a stoma, and enhances bowel continuity in patients with perforated diverticulitis. Several studies also employed negative pressure therapy for temporary abdominal closure. The main prognostic factors identified were ongoing peritonitis and hemodynamic instability, which characterize critically ill patients. These findings underscore the importance of prioritizing patient stability and controlling disease severity at the outset, before proceeding to definitive therapy once the patient is stable. Further high-quality, multicenter studies with larger sample sizes are needed to develop standardized treatment guidelines.

## Introduction and background

Diverticulitis is an inflammatory disease that affects the small pouches, known as diverticula, formed by weak areas, primarily - but not exclusively - of the sigmoid colon. Approximately 150,000 hospital admissions in the United States (US) each year are due to diverticulitis, with about 50,000 resulting in bowel resection [1]. Perforation, a serious complication of this disease, occurs when a tear develops in the walls of the diverticula [2]. A significant cause of diverticulosis is low fiber intake, often attributed to the high consumption of white flour in Western society. This dietary change can reduce stool bulk and prolong gastrointestinal transit time. Slowed colonic motility may increase colonic pressure, leading to the formation of diverticulosis. The most serious complications of diverticulitis can include abscesses, obstruction, fistulas, or perforations. Approximately 5-10% of individuals have diverticula in their colon by around age 40, and this incidence rises to 60-70% by age 80 in the US population [3].

“For he who fights and runs away will live to fight another day, but he who is in battle slain will never rise and fight again,” remarked Oliver Goldsmith [4]. This quote captures the aim of damage control surgery (DCS). DCS is a staged surgical approach that is commonly used in emergency conditions, specifically in abdominal trauma. It prioritizes the physiologic state before anatomic repair. It resembles the warrior who retreats to fight once again, as DCS defers definitive surgery and prioritizes temporary measures to stabilize the patient. DCS is associated with improved mortality and complications in severely injured trauma patients. DCS is a staged approach that aims to control hemorrhage and enteral contamination [5]. It is an aggressive form of resuscitation that aims to improve physiology over anatomic repair. DCS fulfills all the major concerns in emergency surgery, like limited surgical trauma, short duration, and source control [6]. In past years, DCS has frequently been referred to as damage control resuscitation. The emphasis is mainly on initial resuscitation of hypotension with blood products and managing the lethal triad of coagulopathy, acidosis, and hypothermia [7]. There are five components to decision-making in damage control: patient selection and the decision to perform damage control, intraoperative reassessment of laparotomy, resuscitation in the ICU, definitive procedures after returning to the operating room, and finally, abdominal wall reconstruction [8].

Perforated diverticulitis is a life-threatening condition that requires surgical management. It may initially present with complications such as sepsis and generalized peritonitis. DCS offers a potentially life-saving, staged surgical approach in this clinical scenario [9]. Although primarily a treatment modality for trauma cases, emerging studies reveal the use of DCS in perforated diverticulitis. However, there are very few studies on managing patients with diffuse peritonitis secondary to perforated diverticulitis using DCS [10]. The use of DCS is emerging as an acceptable alternative, especially for critically ill patients with perforated diverticulitis complicated by peritonitis and sepsis. Perforated diverticulitis can be treated with DCS, which serves as an alternative to anatomic reconstructions. These reconstructions might increase the risk of complications. Currently, there is no consensus regarding the use of DCS for this condition. DCS is emerging as an alternative to Hartmann’s procedure in patients with peritonitis due to purulent or fecal contaminants in acute diverticulitis. Traditional methods sometimes increase the risk of stoma formation but can potentially enhance quality of life. The presence of a stoma can impair psychosocial and lifestyle challenges and increase long-term healthcare expenses. Therefore, restoring bowel continuity is an essential clinical goal that should be prioritized in treatment. DCS was initially targeted at those with abdominal injuries [11]. Recent studies indicate that DCS is also being utilized for other acute conditions, such as abdominal compartment syndrome and septic shock, that cannot be treated with primary closure [12]. This systematic review aimed to summarize whether DCS offers a more beneficial management plan than traditional surgeries for patients with perforated diverticulitis.

## Review

Methods

This systematic review followed the Preferred Reporting Items for Systematic Reviews and Meta-Analyses (PRISMA) 2020 guidelines [13]. The study was conducted by performing a literature search across five major databases: PubMed, Cochrane Library, Google Scholar, ScienceDirect, and MEDLINE. The search aimed to identify studies that compared DCS with other surgical approaches for managing perforated diverticulitis. The search strategy employed medical subject headings (MeSH) terms and standard keywords, such as “damage control surgery” and “perforated diverticulitis”.

**Figure 1:**
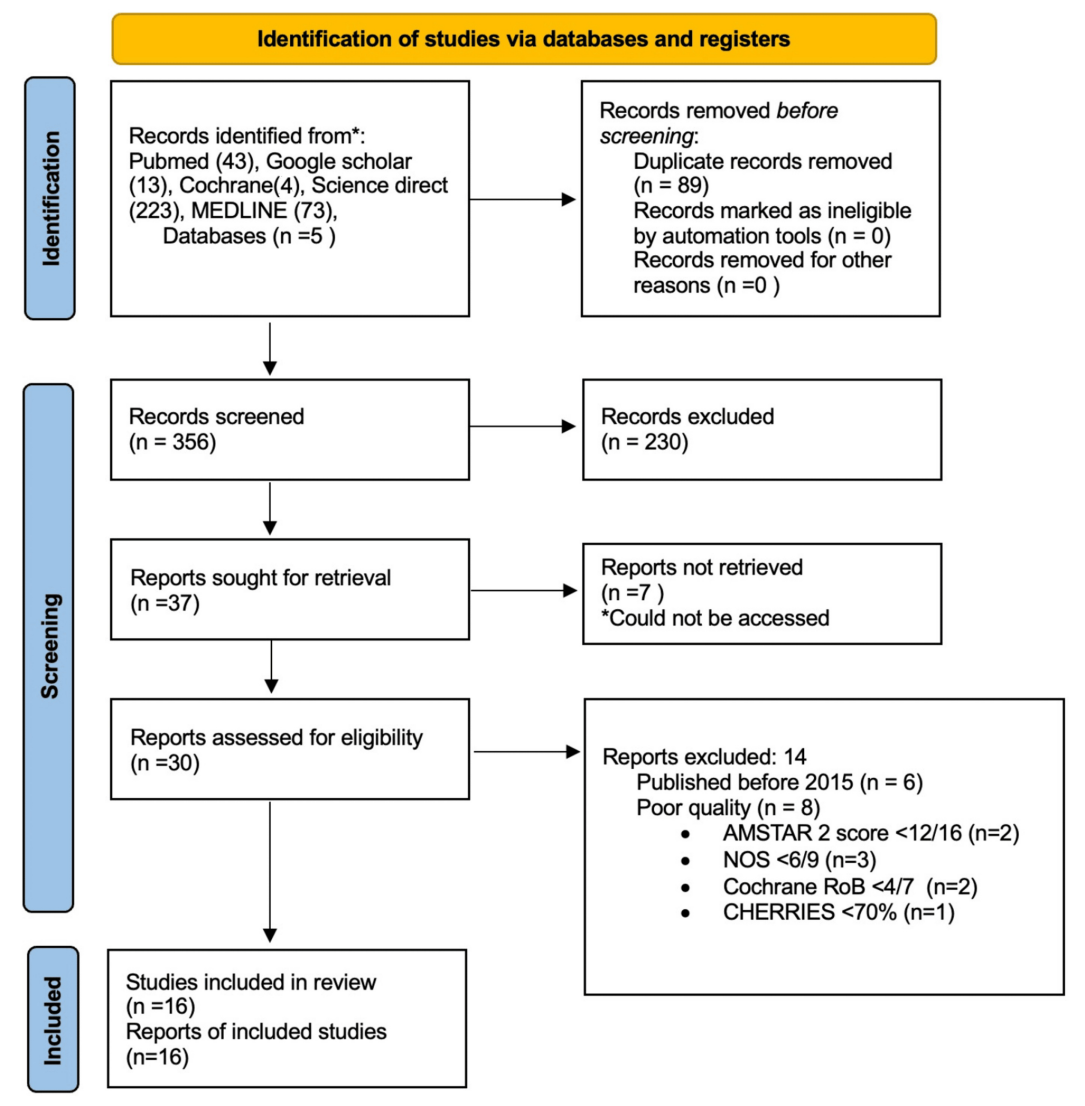
PRISMA flowchart demonstrating the inclusion of selected articles. AMSTAR: Assessment of Multiple Systematic Reviews; NOS: Newcastle-Ottawa Scale; RoB: Risk of Bias; CHERRIES: Checklist for Reporting Results of Internet E-Surveys; PRISMA: Preferred Reporting Items for Systematic Reviews and Meta-Analyses

As shown in Figure 1, the search targeted articles from January 1, 2015, to May 1, 2025. A total of 356 articles were identified across all five databases. These included 43 from PubMed, 13 from Google Scholar, four from Cochrane, 223 from ScienceDirect, and 73 from MEDLINE. After selecting these articles, 89 duplicate entries were removed. Of the remaining, 230 were excluded for not meeting the eligibility criteria. 

The remaining 37 articles were analyzed, of which seven were not retrieved due to lack of access, discontinued journal, or no full text. The other 30 articles, which were full text, were assessed in detail. Fourteen of these were excluded due to their quality or the year of publication being prior to 2015. Six were excluded due to being published before 2015, and eight did not meet the minimum quality appraisal thresholds. The quality appraisal selection criteria are depicted in Table 1. Ultimately, a total of 16 articles were included in this systematic review. 

**Table 1:** Quality appraisal selection criteria. AMSTAR: Assessment of Multiple Systematic Reviews; NOS: Newcastle-Ottawa Scale; RoB: Risk of Bias; CHERRIES: Checklist for Reporting Results of Internet E-Surveys; JBI: Joanna Briggs Institute

Study design	Appraisal tool used	Minimum acceptable score/rating
Systematic review or meta-analysis	AMSTAR 2 [14]	≥12 out of 16
Observational cohort	NOS [15]	≥6 out of 9
Randomized controlled trial	Cochrane RoB [16]	≥4 out of 7 domains (low risk)
Case report	JBI Checklist [17]	≥7 out of 10 domains
Cross-sectional survey study	CHERRIES [18]	≥70% compliance with checklist items

These articles underwent quality appraisals based on their study design, which included tools such as A Measurement Tool to Assess Systematic Reviews 2 (AMSTAR 2) for systematic reviews and meta-analyses, the Newcastle-Ottawa Scale (NOS) for observational studies, the Joanna Briggs Institute (JBI) Checklist for case reports, the Cochrane Risk of Bias (RoB) tool for randomized controlled trials, and the Appraisal of Guidelines for Research and Evaluation II (AGREE II) and the Checklist for Reporting Results of Internet E-Surveys (CHERRIES) for survey guidelines and survey studies, respectively [14-19].

Inclusion and Exclusion Criteria

In this systematic review, we included papers published in the last decade that were available in full text and based on human studies. The included articles contained information on DCS as a treatment for patients with acute perforated diverticulitis. We excluded papers published before 2015, those in foreign languages, and non-human studies to minimize bias and ensure the relevance of the information. Given the limited scientific studies on this topic, we also considered the population size and the scarcity of relevant findings. These criteria, given in Table 2, helped minimize the risk of selection bias and enhance consistency across all analyzed articles.

**Table 2:** Summary of inclusion and exclusion criteria for study selection. DCS: Damage control surgery

Criteria	Category
Inclusion criteria	Full text, English text, human studies from 2015 to 2025
	Article focusing on DCS in perforated diverticulitis
	Any study design
Exclusion criteria	Non-English, animal studies prior to 2015
	Lack of clinical evidence related to DCS in diverticulitis

The review encompassed articles from the past decade that focused on managing perforated diverticulitis with DCS. All articles that were non-English, animal-based, and irrelevant were excluded. This ensured clinical relevance and quality.

Keywords

Table 3 summarizes the keywords used in the search strategy across the five databases.

**Table 3:** Search strategy and keywords used in different databases.

Database	Keywords	Search strategy	Filters	Search results
PubMed	Damage control surgery, perforated diverticulitis	(“Laparotomy”[Mesh] OR Open abdomen OR Surgery OR Laparotomy OR Surgical procedure OR Operative OR General surgery) AND (“Sepsis”[Mesh] OR “Peritonitis”[Mesh] OR “Abdomen, Acute”[Mesh] OR Septic shock OR Sepsis OR Peritonitis OR Acute abdomen) AND (“Diverticulitis”[Mesh] OR diverticulitis OR diverticular disease) AND (damage OR damage control)	Free full text, past 10 years, English	43
Cochrane Library	Damage control surgery, perforated diverticulitis	(diverticulitis OR diverticular disease) in Title Abstract Keyword AND (Septic shock OR Sepsis OR Peritonitis OR Acute abdomen) in Title Abstract Keyword AND (Open abdomen OR Surgery OR Laparotomy OR Surgical procedure OR Operative OR General surgery) in Title Abstract Keyword AND (damage OR damage control) in Title Abstract Keyword	Past 10 years, English	4
Google Scholar	Diverticulitis, damage control surgery, trauma	(Damage control surgery) AND (Perforated diverticulitis)	Past 10 years, English	13
ScienceDirect	Damage control surgery, perforated diverticulitis	(Damage control surgery) AND (Perforated diverticulitis)	Past 10 years, English	223
MEDLINE	Damage control surgery, perforated diverticulitis	(Damage control surgery) AND (Perforated diverticulitis)	Past 10 years, English	73

DCS: ("Laparotomy"[Mesh] OR Open abdomen OR Surgery OR Laparotomy OR Surgical procedure OR Operative OR General surgery) AND ("Sepsis"[Mesh] OR "Peritonitis"[Mesh] OR "Abdomen, Acute"[Mesh] OR Septic shock OR Sepsis OR Peritonitis OR Acute abdomen) AND ("Diverticulitis"[Mesh] OR diverticulitis OR diverticular disease) AND (damage OR damage control).

Diverticulitis: ("Diverticulitis, Colonic/diagnosis"[Mesh] OR "Diverticulitis, Colonic/diagnostic imaging"[Mesh] OR "Diverticulitis, Colonic/mortality"[Mesh] OR "Diverticulitis, Colonic/pathology"[Mesh] OR "Diverticulitis, Colonic/physiopathology"[Mesh] OR "Diverticulitis, Colonic/prevention and control"[Mesh] OR "Diverticulitis, Colonic/radiotherapy"[Mesh] OR "Diverticulitis, Colonic/rehabilitation"[Mesh] OR "Diverticulitis, Colonic/surgery"[Mesh] OR "Diverticulitis, Colonic/therapy"[Mesh] OR "Diverticulitis, Colonic/urine"[Mesh]).

Results

Our study began with an identification process using multiple databases, including PubMed (n=43), Google Scholar (n=13), Cochrane Library (n=4), ScienceDirect (n=223), and MEDLINE (n=73). Out of 356 articles, 89 were duplicates and removed. Following the screening, 230 records were excluded for not meeting the inclusion criteria. The remaining 37 articles were sought for retrieval, of which seven could not be accessed, but the abstracts were screened. They did not meet the inclusion criteria, leaving 30 full-text articles for eligibility screening. Upon reviewing the full text, 14 were excluded: six due to publication before 2015 and eight due to poor methodological quality, assessed using tools like AMSTAR, Cochrane RoB, NOS, and JBI Checklist, among others. Finally, 16 studies met all criteria and were included in this systematic review.

Table 4 presents the quality appraisal of the included studies. A high score on these tools indicates that the studies selected for review meet most of the methodological standards required for reliability, internal validity, and a low risk of bias. Here, the thresholds applied for these tools were AMSTAR 2 (≥12/16), NOS (≥6/9), Cochrane RoB (≥4/7), and CHERRIES (≥70%). A higher score suggested that the methodology used was robust and the articles had appropriate study design and reporting standards. The higher the score, the stronger the conclusion drawn. This method contributed to making this review provide more accurate and effective results in terms of DCS in perforated diverticulitis.

**Table 4:** Quality appraisal of included studies. AMSTAR: Assessment of Multiple Systematic Reviews; NOS: Newcastle-Ottawa Scale; RoB: Risk of Bias; CHERRIES: Checklist for Reporting Results of Internet E-Surveys; AGREE: Appraisal of Guidelines for Research and Evaluation

Study author and year	Study type	Quality appraisal	Score/rating
Tartaglia et al. (2019) [11]	Observational cohort	NOS	7/9
Kafka-Ritsch et al. (2020) [12]	Randomized controlled trial	Cochrane RoB	7/7
Gasser et al. (2019) [20]	Observational cohort	NOS	8/9
Cirocchi et al. (2021) [21]	Systematic review and meta-analysis	AMSTAR 2	13/16
Lambrichts et al. (2019) [22]	Randomized controlled trial	Cochrane RoB	5/7
Nascimbeni et al. (2021) [23]	Multidisciplinary position paper	AGREE II	High
Brillantino et al. (2019) [24]	Observational cohort	NOS	8/9
Sohn et al. (2016) [25]	Observational cohort	NOS	8/9
Sohn et al. (2018(i)) [26]	Observational cohort	NOS	8/9
Sohn et al. (2018(ii)) [27]	Observational cohort	NOS	8/9
Vennix et al. (2016) [28]	Propensity-matched cohort	Cochrane RoB	4/7
Galbraith et al. (2017) [29]	Meta-analysis	AMSTAR 2	13/16
Schmidt et al. (2018) [30]	Meta-analysis	AMSTAR 2	12/16
Gachabayov et al. (2018) [31]	Systematic review and meta-analysis	AMSTAR 2	12/16
Sohn et al. (2021) [32]	Observational cohort	NOS	6/9
Gutiérrez Delgado et al. (2022) [33]	Case report	JBI Checklist	8/10

The selected final 16 studies included various study designs as summarized in Table 5.

**Table 5:** Summary of final studies by study design and appraisal tools used. AMSTAR: Assessment of Multiple Systematic Reviews; NOS: Newcastle-Ottawa Scale; RoB: Risk of Bias; CHERRIES: Checklist for Reporting Results of Internet E-Surveys; JBI: Joanna Briggs Institute; AGREE: Appraisal of Guidelines for Research and Evaluation

Study design	Number of studies	Appraisal tool	Study citation
Randomized controlled trials	2	Cochrane RoB	[12,22]
Systematic reviews and meta-analyses	4	AMSTAR 2	[21,29-31]
Observational cohorts	8	NOS	[11,20,24-28,32]
Case reports	1	JBI Checklist	[33]
Cross-sectional surveys and guidelines papers	1	CHERRIES/AGREE II	[23]

Table 6 highlights the importance of DCS in unstable patients with perforated diverticulitis, where early mortality reduction is critical.

**Table 6:** Summary table with relevant findings of final studies. NPWT: Negative pressure wound therapy; DCS: Damage control surgery; ↓: Decreased; ↑: Increased

Study author and year	Type of study	Sample size (if applicable)	Findings in DCS
Mortality			
Kafka-Ritsch et al. (2020) [12]	Randomized controlled trial	21	↓ mortality
Tartaglia et al. (2019) [11]	Observational cohort	34	12% mortality and feasible DCS
Gasser et al. (2019) [20]	Observational cohort	78	Mortality of 3% in NPWT alone vs 7% for NPWT with additional transparent drape
Ciroochi et al. (2021) [21]	Systematic review and meta-analysis	3	9.2% mortality
Sohn et al. (2016) [25]	Observational cohort	37	8.2% mortality
Sohn et al. (2018(ii)) [27]	Multicenter observational	58	9% mortality
Sohn et al. (2021) [32]	Observational cohort	122	8.2% mortality
Sohn et al. (2018(i)) [26]	Observational cohort	74	Higher mortality, stoma, and complications if accompanied by ongoing peritonitis
Stoma rates			
Kafka-Ritsch et al. (2020) [12]	Randomized controlled trial	21	↓ stoma
Tartaglia et al. (2019) [11]	Observational cohort	34	↓ stoma
Brillantino et al. (2019) [24]	Observational cohort	30	77% stoma-free survival
Sohn et al. (2016) [25]	Observational cohort	37	↓ stoma
Sohn et al. (2018(ii)) [27]	Multicenter observational	58	50% stoma rate
Galbraith et al. (2017) [29]	Meta-analysis	372	↓ stoma
Vennix et al. (2016) [28]	Cohort	177	↑ stoma reversal (laparoscopic 88% vs open 62%)
Schmidt et al. (2018) [30]	Meta-analysis	1223	↑ stoma reversal in primary anastomosis
Bowel continuity and anastomosis			
Kafka-Ritsch et al. (2020) [12]	Randomized controlled trial	21	↑ continuity
Tartaglia et al. (2019) [11]	Observational cohort	34	71% continuity
Ciroochi et al. (2021) [21]	Systematic review and meta-analysis	318	62.1% continuity
Sohn et al. (2016) [25]	Observational cohort	37	77% anastomosis
Sohn et al. (2018(ii)) [27]	Multicenter observational	58	83% anastomosis
Sohn et al. (2021) [32]	Observational cohort	122	77% anastomosis
Gachabayov et al. (2020) [31]	Meta-analysis	1016	↑ continuity with primary anastomosis

Discussion

DCS is a procedure such as an initial abbreviated laparotomy for rapid source control, temporary abdominal closure techniques such as negative pressure wound therapy (NPWT), ICU resuscitation to reverse acidosis, hypothermia, and coagulopathy, and planned re-laparotomy for definitive repair, which may include primary anastomosis (PRA) or stoma after physiologic stabilization.

The use of DCS in managing perforated diverticulitis has gained increasing attention over the past decade, particularly in critically ill patients with generalized peritonitis and sepsis. Unlike conventional single-stage approaches, DCS focuses on the immediate management of the source and the physiological stabilization of the patient before definitive surgical intervention. This review consolidated the available evidence from the past 10 years on the efficacy, safety, and outcomes associated with DCS. This discussion seeks to elucidate the emerging role of DCS, its comparative advantages, and its implications for surgical decision-making in managing acute perforated diverticulitis.

**Figure 2:**
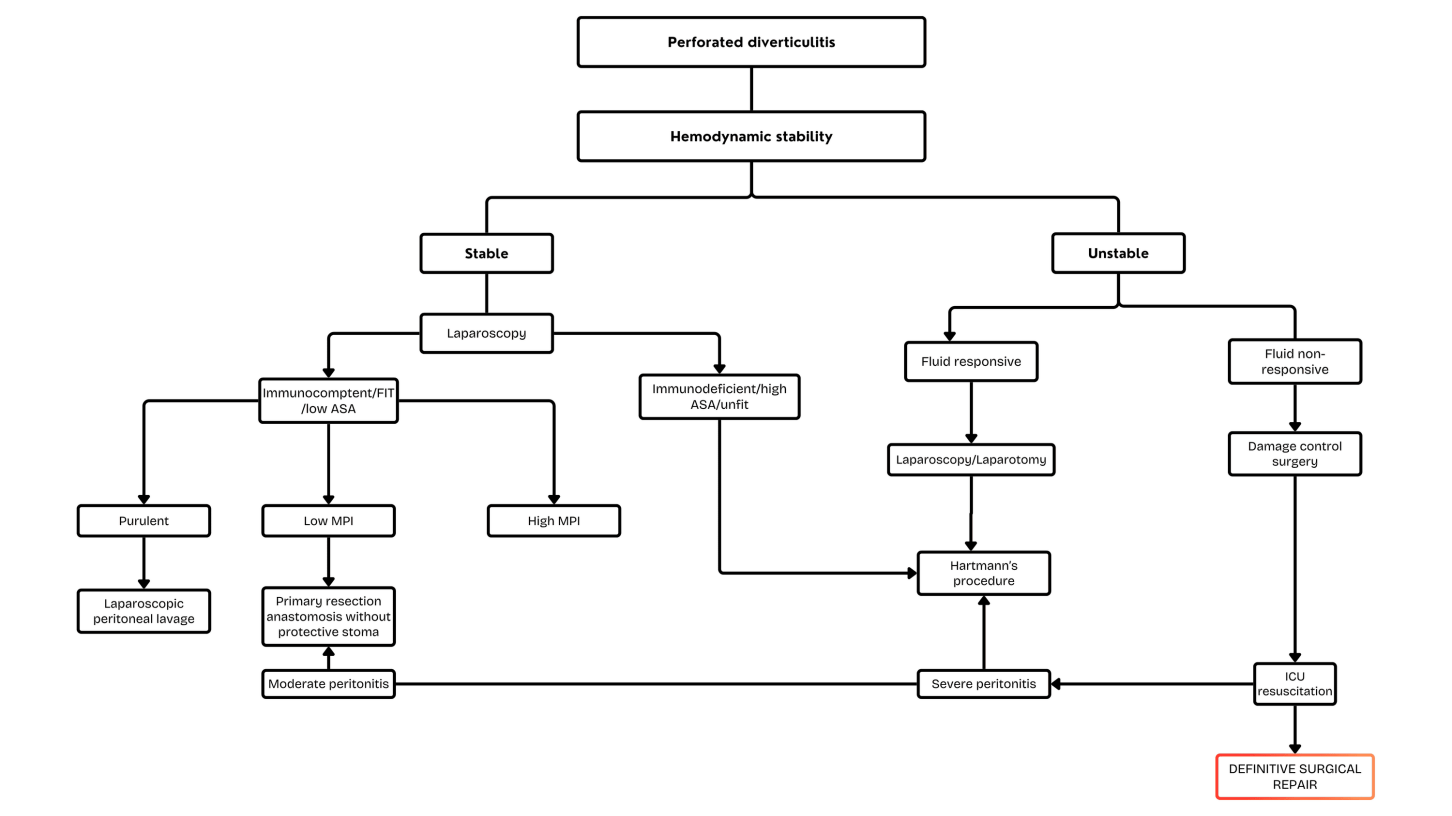
Stepwise management algorithm for patients with peritonitis. PRA: Primary anastomosis; MPI: Mannheim Peritonitis Index; ASA: American Society of Anesthesiologists The figure was created by the author Samyuktha Harikrishnan based on [20].

Figure 2 illustrates the management plan for perforated diverticulitis in a stepwise manner. The management of patients with DCS depends upon the patient’s hemodynamic status, immunologic status, and severity of peritonitis. Those who are stable are initially assessed with laparoscopy. If they are immunocompetent and have a low American Society of Anesthesiologists (ASA) score, then management is based on another score called the Mannheim Peritonitis Index (MPI). The ASA score is a scoring system developed by the American Society of Anesthesiologists (ASA). The ASA score helps assess the preoperative status of patients, enabling informed treatment decisions. A low ASA score points toward healthy patients or those with mild systemic disease. These patients will be able to tolerate more extensive surgeries, such as PRA. A high ASA score indicates that patients with extensive disease can benefit from DCS. The MPI is a scoring system that predicts the risk of mortality based on age, organ failure, and extent of contamination. Those with purulent peritonitis without perforation can undergo laparoscopic lavage, while those with low MPI scores should undergo PRA without a protective stoma [20,34,35].

Clinical Implications

In comparison, those who are immunodeficient with a high ASA score are managed with Hartmann’s procedure. Unstable patients should be resuscitated with fluids and can be subdivided into fluid-responsive or non-responsive groups. Those who are responsive can be managed with laparotomy or laparoscopy, while those with severe peritonitis and non-responsive to fluids are managed with Hartmann’s procedure. Those who are non-responsive to fluid are commonly due to septic shock and are the leading candidates for DCS. This prioritizes source control with temporary closure of the abdomen, followed by transfer to the ICU for resuscitation. Once the patient is stabilized in the ICU, they can undergo definitive staged repair [23].

Feasibility, Safety, and Mortality

DCS is one of the ideal management plans for patients with diverticulitis with generalized peritonitis or instability. The main principle of DCS is to prioritize source control over definitive resection. This helps halt the downward spiral of physiologic deterioration by prioritizing the management of coagulopathy, hypothermia, and acidosis, which allows for resuscitation and stabilization. Following this, patients undergo a definitive surgical repair. PRA is primarily reserved for stable patients who can tolerate this procedure. The use of DCS can help bridge the gap and manage unstable patients by maintaining low complication rates and improving safety [21].

Several studies demonstrate reduced complication rates and 30-day mortality. A randomized controlled trial by Kafka-Ritsch et al. (2020) showcased that DCS in patients with perforated diverticulitis enhanced bowel reconstruction and reduced stoma rates at discharge [12]. Similarly, Tartaglia et al. (2019) reported improved mortality and reduced stoma rates in patients undergoing staged DCS [11]. The review conducted by Cirocchi et al. (2021) depicted that around 62.1% achieved bowel continuity by the DCS method. DCS was used for generalized peritonitis secondary to complicated acute diverticulitis with serious leaks. A continuity rate of 4.7% and an overall mortality of 9.2% were achieved. This study also revealed other findings, such as a low rate of septic shock. This was likely due to selection bias resulting from the lack of reported cases. The authors suggested a tailored approach to managing patients based on the severity of the case [21].

Lambrichts et al. (2019) conducted a large randomized controlled trial to compare Hartmann’s procedure with PRA in patients with fecal and purulent peritonitis. The findings showed that PRA was a superior management plan compared to Hartmann’s procedure in terms of post-reversal morbidity. Short-term mortality and morbidity were unaffected after the index procedure. They concluded that PRA should be the method of choice for treating perforated diverticulitis in hemodynamically stable and immunocompetent patients [22]. These findings were complemented by another multidisciplinary paper by Nascimbeni et al. (2021), who recommended the use of DCS in fecal and purulent peritonitis secondary to perforated diverticulitis. This conclusion was derived based on clinical severity scoring and systemic compromise [23].

The studies collectively demonstrate the strategic importance of DCS in improving survival rates while also considering the physiologic approach to managing critically ill patients with perforated diverticulitis.

Stoma Rates and Restoration of Continuity

The most critical goals of DCS are to reduce the rates of ostomies and ensure the continuity of the bowel. Observational studies, such as Brillantino et al. (2019), suggest that DCS with NPWT and installation is a beneficial approach in peritonitis caused by perforated diverticulitis. It is associated with low morbidity, stoma rates, and high continuity, making it substantial for both critical and non-critical patients [22]. This finding was also substantiated by Kafka-Ritsch et al. (2020), who found reduced ostomies compared to performing surgical strategies such as Hartmann’s procedure or PRA [12].

Gasser et al. (2019) employed two methods of DCS in patients with perforated sigmoid diverticulitis and found that DCS with negative pressure therapy is a safe and feasible management option. Negative pressure therapy aids in temporary abdominal closure and resuscitation [20].

Sohn et al. have made significant contributions over the years, showcasing DCS in the management of perforated diverticulitis through their studies from 2016 to 2021. In their 2016 and 2018(i) studies, 84% reported anastomosis with successful restoration of bowel continuity [25,26]. In their 2018(ii) multicenter study, they identified ongoing peritonitis (OP) as a strong marker of poor outcomes. Those with OP had higher rates of morbidity (44% vs 24%), mortality (12% vs 0%), and enterostomy at discharge (76% vs 36%) in comparison to those without OP [27]. These findings underscore the importance of intraoperative assessment and staging in perforated diverticulitis.

Gasser et al. (2019) provided evidence that the approach of staged decision-making helps reduce the potential need for a permanent ostomy [20]. Vennix et al. (2016) conducted a propensity-matched cohort study that showed favorable outcomes with a staged approach. This, in turn, allowed for safely delayed anastomosis [28]. A meta-analysis that focused on laparoscopic lavage for Hinchey III diverticulitis concluded that there was no evidence of increased mortality compared to colon resection. Laparoscopic lavage was found to have lower rates of stoma and a higher rate of reoperation. Additionally, the infection rates were higher with lavage compared to colon resection [29].

Furthermore, other studies, such as Schmidt et al. (2018), a systematic review with evidence, suggested a higher perioperative mortality risk but a lower stoma reversal rate for PRA compared to Hartmann’s procedure. They also indicated that laparoscopic lavage is not superior to primary resection for Hinchey III perforated diverticulitis [30]. Another meta-analysis highlighted that restorative resection and staged management are associated with better continuity. The authors recommended PRA with diverting ileostomy in stable patients with complicated perforated diverticulitis [31].

The findings in these studies highlight the ability of DCS to reduce stoma rate dependency and enhance the continuity and functioning of the bowel in patients with perforated diverticulitis.

Therapeutic Outcome and Prognostic Factors

The most recent study by Sohn et al. (2021) found an 8.2% 30-day mortality with a 77% rate of anastomosis. This demonstrates that despite being outside a specialized center, DCS is a safe and effective management plan for perforated diverticulitis [32].

In addition to observational studies, a particular case report also provides evidence. Gutiérrez Delgado et al. (2022) reported the usefulness of DCS in retroperitoneal perforated diverticulitis complicated by myositis. They attempted a staged approach that prioritized sepsis control and source management and delayed definitive repair by stabilizing this patient. This case highlights the compatibility of DCS in managing one of the worst complications of perforated diverticulitis [33].

Conventional management, such as Hartmann’s procedure or primary repair and anastomosis for index surgery, represents a different approach to managing perforated diverticulitis. It is reserved explicitly for physiologically unstable patients who cannot tolerate an extensive initial procedure. Although traditional management aims to do a single-stage surgery as a definitive treatment, DCS focuses on managing the physiologic status, followed by resuscitating the patient in the ICU. Once the patient is stable in the ICU, a definitive treatment, including a second surgery, can be performed.

These studies provide strong confirmation of the use of DCS as a lifesaving approach in perforated diverticulitis. Waiting to perform definitive treatment until the patient is stable improves short-term results and helps restore bowel continuity, the primary goal of modern surgical care.

Limitations

Multiple limitations were acknowledged during this review. There was limited generalizability due to the modest sample size across various studies, as most focused on a single center. A meta-analysis could not be conducted due to the heterogeneity in the study design. Since the selected studies were only full-text articles in English, there was a selection bias. The studies included exhibited significant heterogeneity in study design and patient population. Due to this reason, the feasibility of conducting a meta-analysis was limited, preventing the pooling of effects. 

Future Directions

DCS is appropriate for specific cases of perforated diverticulitis, particularly among unstable patients, and is progressively becoming the standard clinical practice. Future research should aim to enhance patient selection criteria and tailor surgical protocols to individual cases to improve patient outcomes. Multicenter RCTs are necessary to compare DCS with alternative procedures such as Hartmann’s procedure and PRA. Furthermore, additional research focused on quality of life, stoma reversal rates, and both short-term and long-term outcomes is essential. Such studies will offer valuable insights into patient-centered care and help develop standardized protocol guidelines.

## Conclusions

This review mainly focused on the role of DCS in managing patients with perforated diverticulitis, specifically those with physiologic instability or generalized peritonitis. This review included 16 studies, which were a combination of randomized controlled trials, cohorts, systematic reviews, and meta-analyses. When DCS is incorporated into severe conditions of perforated diverticulitis, it improves survival, reduces early morbidity, and increases the chance of bowel continuity, which is the ultimate aim. With the emerging use of DCS, other traditional approaches, such as Hartmann’s procedure or PRA, are being reduced. This review shows a downward trend in stoma rates and an increase in anastomoses facilitated by staged interventions. The success of this method relies on evaluating each case separately and following staged surgical principles. Future studies in this field should aim to include a larger sample size and use a standardized scoring system for patients with ASA or MPI classifications. This scoring system will help establish clear and cost-effective guidelines. Integrating surgical criteria and evaluation of long-term prognosis that includes quality of life and stoma reversal rates will guide more personalized and evidence-based management strategies in those with perforated diverticulitis. In the long run, DCS is a promising approach, specifically in high-risk and resource-limited situations.
